# Simulation Analysis of the Semi-Trailer Steered Wheels Control Algorithm

**DOI:** 10.3390/s25030626

**Published:** 2025-01-22

**Authors:** Michał Abramowski, Piotr Fundowicz, Hubert Sar, Andrzej Reński, Mateusz Brukalski

**Affiliations:** Institute of Vehicles and Construction Machinery Engineering, Warsaw University of Technology, 84 Narbutta Str., 02-524 Warsaw, Poland; piotr.fundowicz@pw.edu.pl (P.F.); hubert.sar@pw.edu.pl (H.S.); andrzej.renski@pw.edu.pl (A.R.); mateusz.brukalski@pw.edu.pl (M.B.)

**Keywords:** semi-trailer control, semi-trailer wheel control algorithm, steered wheels, truck tractor with steered wheels, swept path width

## Abstract

As part of improving road safety around trucks, a solution was proposed to reduce the swept path width of a moving tractor–semi-trailer. This article presents a mathematical analysis of the movement of a tractor unit with a traditional semi-trailer with fixed axles and steered wheels. A simulation analysis of both presented vehicles was carried out. The core of the algorithm controlling the steering angle of the semi-trailer wheels is presented. The influence of controlling the semi-trailer’s swivel wheels on the swept path width of a tractor–trailer with a semi-trailer equipped with swivel wheels is discussed. The assumptions for building the control algorithm are presented. The article presents the advantages of the solution used along with the control algorithm. Measurable benefits resulting from the use of the presented solution are presented, such as increasing cargo space, reducing cargo transport costs, and reducing aerodynamic resistance and fuel consumption. It is worth emphasizing that reducing fuel consumption is very important because it reduces the emission of harmful exhaust gases into the atmosphere. The swept path width is important especially in the case of vehicles moving in a limited area, for example in the parking lots of transhipment and logistics centers, between urban buildings. Vehicles admitted to traffic meet the minimum conditions imposed by homologation regulations, but reducing the swept path width allows for improving the operational properties of the tractor–semi-trailer. The use of the proposed control algorithm to control the turn of the semi-trailer’s steered wheels brings tangible benefits both in improving road safety and in reducing the emission of harmful substances into the environment.

## 1. Introduction

There are many vehicles on the road, including trucks. Commercial vehicles can be subdivided by payload, by size, and also by intended use [1,2]. However, the most commonly used vehicle types are lorries with trailers or tractor units with semi-trailers. The main disadvantage of a lorry with a trailer is the type of body that is fitted to the vehicle. Most bodies are permanently fitted, which makes it impossible to radically change the type of load carried by these vehicles (bulk materials and liquids). There are solutions offered that allow a quick change of the type of bodywork [3], e.g., BDF, but such a solution reduces the load capacity of the vehicle and increases the bodywork and operating costs of these vehicles. In trucks with trailers, loading itself is also often problematic, as the trailer has to be loaded separately and the truck separately as two different vehicles. A tractor unit with semi-trailer is a more universal vehicle. If you want to change the type of load you are carrying, you simply change the semi-trailer. The tractor unit remains the same and can be used to move different types of material. A semi-trailer tractor is also less difficult to load. The semi-trailer is one large loading area that is easier to move around in. One of the biggest disadvantages of a semi-trailer tractor is the large swept path width in which this vehicle moves, especially if the movement is along a curve with a small radius. As a result, it is very difficult or completely impossible to drive a tractor–semi-trailer on local roads or negotiate small intersections with circular traffic.

There are many works which discuss the problems of semi-trailer motion. For example, in the article [4], the active safety of semi-trailers is investigated with the application of electronically controlled pneumatic braking systems (ECPBSs).

Another work, [5], discusses the issues concerning the risks to goods transported by semi-trailers on highways, including their motion dynamics.

In the work [6], the authors present the problems of the differential brake control of self-driving tractors with semi-trailers with the application of an advanced motion model, thus showing how to control the trajectory tracking of a tractor with a semi-trailer.

In the paper [7], there is presented an investigation of a tractor–semi-trailer set with the application of a road simulator including road obstacles. The work consists of both a test rig experiment as well as numerical calculations.

In the article [8], it is assessed how selected parameters of tractor–semi-trailer sets influence the indicators of braking maneuvers. The role of anti-lock brake systems is also presented in this paper.

The paper [9] discusses the significance of the state of the load of a vehicle transporting cargo and the type and the state of the road surface a vehicle is moving on.

In the article [10], there is presented a numerical investigation of liquid tank semi-trailers.

In the next work, [11], there is presented a complex model of vehicles’ motion, including, among others, tractor–semi-trailer sets. The author included here the unevenness of the road surface and the internal and external excitations of a vehicle.

The article [12] discusses the problem of braking timing sequence and its significant impact on the stability of braking a semi-trailer train. To perform the simulation, the TruckSim numerical tool was applied by the authors.

In the work [13], there discussion of the issue of lateral stability control in cases of tractor–semi-trailers moving at high velocity. A five-degree-of-freedom model was applied in this paper, thus enabling the investigation of the yaw–roll dynamics of a vehicle.

The authors of the article [14] discuss the problematic nature of the blind zone which takes place during the right-turn maneuvers of semi-trailer trucks.

In the elaboration [15], the authors are focused on the issue concerning the influence of dynamic load reduction through the air suspension system of the axles of the semi-trailer.

There can be also found in the literature works regarding SPA—swept path analysis—for the evaluation and calculation of the (3D) space required when an oversized vehicle engages in turning maneuvers. One example of an approach to this problem is the article [16], in which the authors include their algorithm for transient offtracking analysis. Another work regarding a similar issue is the paper [17], in which swept-path analysis was included for the design of turbo-roundabouts, including the theory of vehicle motion geometry. A topic similar to the one presented in [17] can be found in the paper [18], where the swept-path analysis was included in designing single-lane roundabouts. Swept-path analysis can be also found in the case of the article [19], where it is included for the design of roads in the mountains. Analogical analysis was applied in the work [20] regarding suburban roundabouts.

This article presents a way of eliminating this disadvantage of tractor–trailer units with semi-trailers. Using the solution presented in the article also makes it possible to increase the load space of the semi-trailer or to increase the strength of the front wall of the semi-trailer against the impact of the moving load. An analysis and comparison of tractor–trailer units with conventional semi-trailers and tractor–trailer units with semi-trailers with the proposed design change to the semi-trailer chassis is presented. Computer simulations have also been carried out to show how much the swept path width can be reduced.

Reducing the swept path width may improve the safety associated with the operation of a tractor–semi-trailer, especially in closed areas such as logistics centers, transport bases or transhipment centers.

### The Role of Ultrasonic Sensors and Image Processing in Investigating a Vehicle Towing a Trailer

In this subchapter of the article, there are cited the following works, based on ultrasonic sensors or the algorithms based on image processing.

In the work [21], the authors discuss the problem of maneuverability and stability of three-link road trains type (the so-called “b-triple”) with steered axles of semi-trailer dollies. The authors mention here the dual drive control in the case of steered axles on semi-trailer dollies.

In the work [22], there is presented the four real-time implementable nonlinear model predictive control (NMPC) formulations with the use of the hitch angle measurement for the torque-vectoring (TV) control of an electric automobile which is towing a trailer. A dynamic model of a trailer is mentioned in the article [22].

There are some possibilities regarding the application of ultrasonic sensors or the systems based on image processing as described in the papers [23,24,25].

Section 2, entitled Method, presents a geometric analysis of the tractor–semi-trailer in the classic version and after the modification proposed by the authors.

Section 3 discusses the obtained calculation results based on the calculation results presented in Section 2.3 of this work.

Section 4, titled Conclusions, refers to the obtained results and indicates how important modifications involving the introduction of a steering axle in a truck semi-trailer may be.

## 2. Method

**Figure 1:**
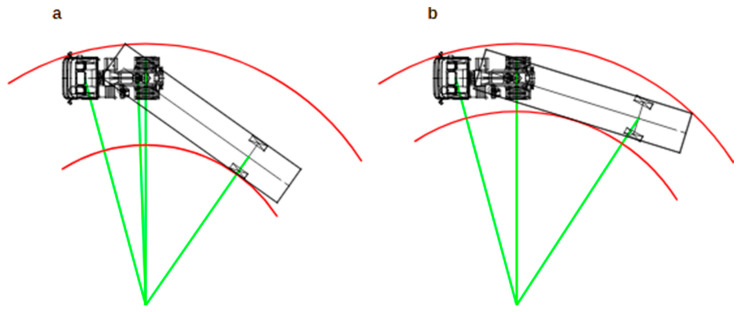
Turning of a typical combination vehicle (**a**) and a combination with a semi-trailer with a steering axle (**b**).

Figure 1 shows a steering diagram of a typical combination vehicle (Sketch a) and a combination with a semi-trailer with a steering axle (Sketch b) in steady motion. In both cases, the same steering angle of the tractor wheels is assumed, resulting in movement on the same track, with the same radius and obtaining the same turning diameter of the tractor.

If the wheels of the semi-trailer turn, reducing the angle of the combination vehicle can result in a smaller dynamic swept path width through which the combination vehicle travels. It may also be advantageous to increase the radius of movement of the center of gravity of the semi-trailer due to the inertial forces (centrifugal force) in the curved movement. However, this is not the subject of the study.

For a determined tractor movement, the outer turning radius can be determined, which is the radius of the track of the outermost point. Assuming the same turning center for the semi-trailer as for the tractor, the steering angle of the combination vehicle can be determined. This is the angle between the longitudinal axis of the tractor and the longitudinal axis of the semi-trailer, at which the outermost point of the semi-trailer will follow the same track as the outermost point of the tractor. Knowing the angle of inflection of the combination vehicle and the center of curvature of the track, the steering angle of the semi-trailer can be determined. In this way, the steering angle of the semi-trailer is not directly dependent on the steering angle of the tractor, which can be varied freely by the driver, but depends directly on the traffic conditions, i.e., the actual turning of the combination vehicle.

The case of transient traffic, when the radius of the curve and the angle of the bend of the tractor–semi-trailer combination changes, is not described in this article.

### 2.1. Analysis

A two-axle tractor and a single-axle semi-trailer have been adopted for the analysis. According to the rules for determining the center of rotation of a vehicle, if the motion of a three-axle tractor (or one with more than three axles) is analyzed, the point adopted in the analysis as the center of the rear axle is the point at the geometric center, between the rear axles of the tractor. Similarly, in the case of a semi-trailer with more than one axle, the point adopted in the analysis as the center of the axle is the point at the geometric center, between the axles of the semi-trailer that are involved in its movement (not lifted).

In the analysis, the outline of the tractor and the outline of the semi-trailer have been replaced by rectangles.

A simplification has been adopted whereby the steering angles of the wheels of the same axle (tractor and semi-trailer) are equal.

**Figure 2:**
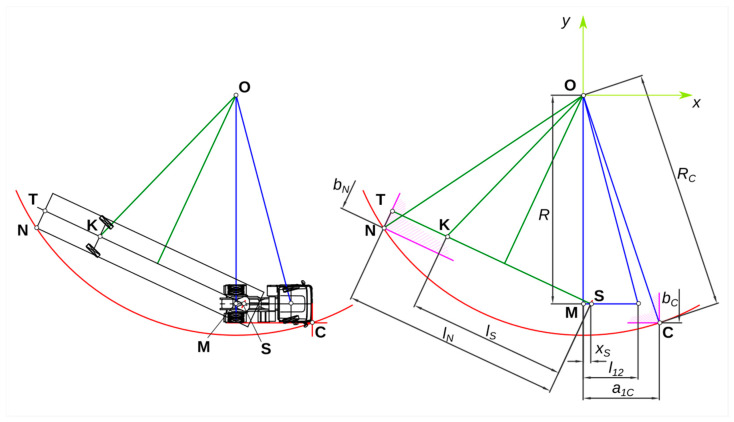
Basic markings and dimensions of the combination vehicle when turning—description in the text (dark blue refers to the tractor, green to the semi-trailer, violet to parts of the vehicle outline).

The most important dimensions of the combination vehicle that are taken into account in the analysis are thus (Figure 2):

*l*_12_—tractor wheelbase,
*a_*1*C_*—distance from the rear axle of the tractor to the front of the tractor,
*x_S_*—distance from the rear axle of the tractor to the center of the coupling (fifth wheel),
*b_C_*—tractor width,
*l_N_*—distance from the rear of the semi-trailer to the center of the coupling (fifth wheel),
*l_S_*—distance from the centerline of the semi-trailer to the center of the coupling (fifth wheel),
*b_N_*—width of a semi-trailer.

The motion of the combination vehicle is analyzed on a horizontal plane. The position of the combination vehicle is described in a coordinate system with its origin at the tractor’s center of rotation—point “O” in Figure 2. The instantaneous position of the tractor in the coordinate system, in which its longitudinal axis is parallel to the abscissa axis (*x*-axis), was assumed for the analysis.

The individual designations of the characteristic points:

*O*—turning center of the tractor and, in the case of stationary movement, of the whole combination vehicle,
*M*—center of rear axle of the tractor,
*S*—center of the coupling (fifth wheel),
*C*—the outermost point of the tractor outside the track; replacing the silhouette of the tractor in the plan view with a rectangle, this is the front outer corner of the tractor,
*T*—point lying on the longitudinal axis of the semi-trailer on its rear outline,
*K*—center of semi-trailer axle,
*N*—the outermost point of the semi-trailer on the track; replacing the silhouette of the semi-trailer in the horizontal projection with a rectangle, this is the rear external corner of the semi-trailer.

It is assumed that in a steady state of curved movement, the tractor wheels are turned at an angle *ϑ_C_*, the steering angle of the semi-trailer wheels is *ϑ_N_*, and the angle between the longitudinal axis of the tractor and the longitudinal axis of the semi-trailer (angle of kink of the combination vehicle) is *α*. Ignoring the drift angles of the individual wheels (assuming, for example, low-velocity motion), the tractor’s turning radius, measured from the turning center point *O* to the center of the tractor’s rear axle *M*, is:

(1) $$R=\frac{l_{12}}{tg\cdot \vartheta_{C}}.$$

The track radius of the front outside corner of the tractor is:

(2) $$R_{C}=\sqrt{{\left(R+\frac{1}{2}\cdot b_{C}\right)}^{2}+a_{1C}^{2}}.$$

The coordinates of the center point of the rear outline of the semi-trailer (point “*T*”) at the optimum angle of kink of the combination vehicle α in the adopted coordinate system (with origin at point “*O*”—Figure 2) are:

(3) $$x_{T}=x_{S}-l_{N}\cdot cos\alpha ,$$

(4) $$y_{T}=R_{C}-l_{N}\cdot sin\alpha .$$

The coordinates of the point at the outer rear corner of the semi-trailer (“*N*” point) at the optimum combination vehicle angle *α* (such that the tractor and semi-trailer have the same external turning radius) are:

(5) $$x_{N}=x_{T}-\frac{1}{2}\cdot b_{N}\cdot sin\alpha ,$$

(6) $$y_{N}=y_{T}-\frac{1}{2}\cdot b_{N}\cdot cos\alpha .$$

The radius of the track of the outer rear corner of the semi-trailer at the optimum steering angle of the combination vehicle *α* is:

(7) $$R_{N}=\sqrt{x_{N}^{2}+y_{N}^{2}}.$$

The value of the optimal angle of refraction α of the assembly is determined by solving the equation:

(8) $$R_{C}=R_{N}.$$

Knowing the steering angle of the combination vehicle, the optimum steering angle of the semi-trailer wheels can be determined from the geometrical relationships:

(9) $$\vartheta_{N}=arctg\frac{l_{S}\cdot sin\alpha -R}{x_{S-}-l_{S}\cdot cos\alpha }+\alpha -90{}^{\circ}.$$

### 2.2. Assumptions Used for the Calculations

Calculations have been made based on exemplary tractor data:

- wheelbase: *l_12_* = 5.0 m,
- width: *b_c_* = 2.55 m,
- distance from rear axle to front of tractor: *a_*1*c_* = 6.50 m,
- distance from rear axle to fifth wheel: *x_s_* = 0.5 m and semi-trailer data:
  - distance from coupling pin to rear outline: *l_n_* = 15.0 m,
  - width: *b_n_* = 2.55 m,
  - rear overhang: *a_2n_* = 3.0 m, so the distance from the fifth wheel to the rear axle *l_s_* = *l_n_*—3.0 m = 12.0 m

### 2.3. Results

**Figure 3:**
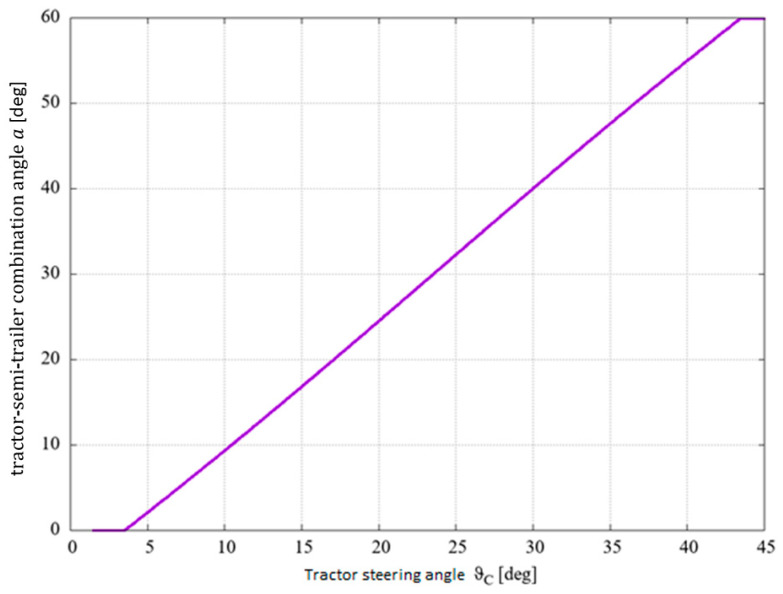
Relationship between the steering angle of the tractor and the steering angle of the combination vehicle for exemplary data.

**Figure 4:**
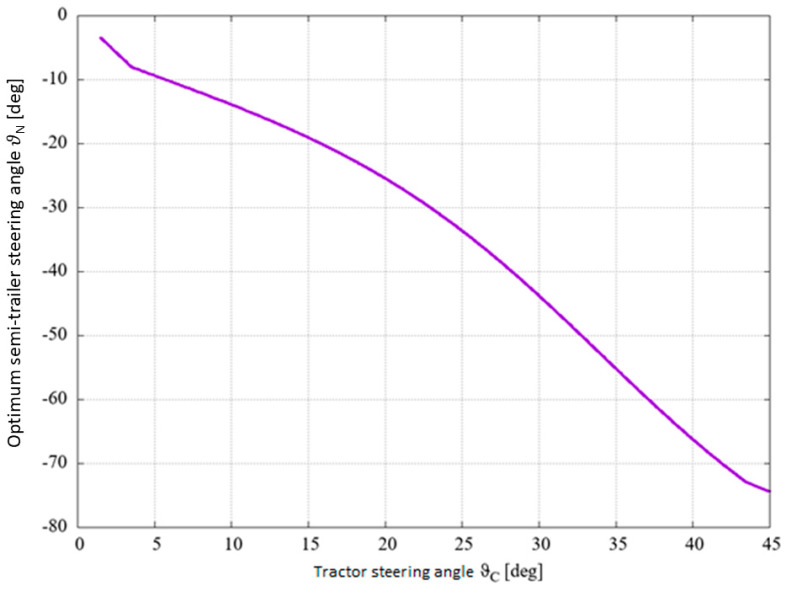
Relationship between tractor steering angle and optimum semi-trailer steering angle for sample data.

The following diagrams show the relationship between the steering angle of the tractor and the steering angle of the combination vehicle (Figure 3) and the relationship between the steering angle of the tractor and the optimum steering angle of the semi-trailer (Figure 4).

**Figure 5:**
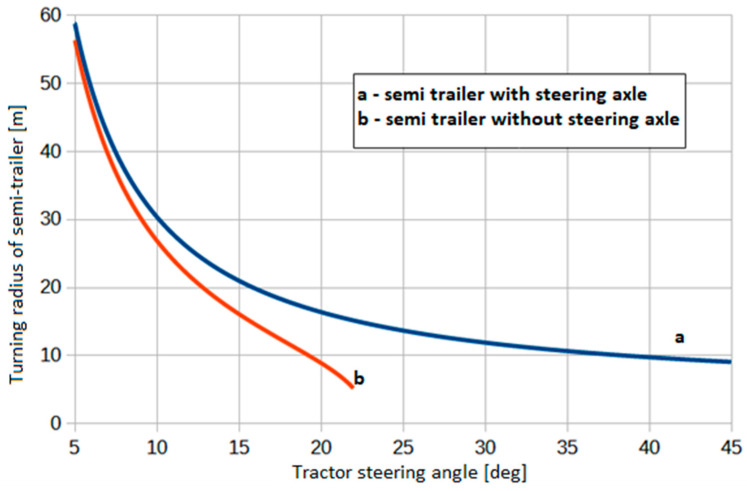
Difference between the turning radius of a semi-trailer without and with steered axle.

The difference between the turning radius of a typical semi-trailer (without steering axle) and a semi-trailer whose wheels are turning according to the described algorithm is shown in the diagrams in Figure 5. A significant difference appears at large steering angles of the tractor, for example, those under consideration—above about 15–20°. It is worth noting that, when turning a combination vehicle with a semi-trailer without turning wheels, the angle of the combination vehicle is limited by its geometry. For a combination vehicle with the data from the example, it is only possible to turn with a tractor steering angle of up to approximately 23°. With this turn, the semi-trailer axle rotates around the center of the semi-trailer axle. This is a purely theoretical situation; the steering angle of the combination vehicle is also limited by the condition of no collision between the semi-trailer and the tractor cab.

## 3. Results and Discussion

Typical semi-trailers used for transport have axles with fixed—not steered—wheels. Some semi-trailers, which are usually used to transport especially heavy loads, use steering wheel axles or axles mounted on turnover shafts without steering angle control [26]. Depending on the traffic conditions—the radius of the curve over which the combination vehicle is moving—the wheels align themselves in the direction resulting from the system of forces acting on them.

The idea behind the solution described in this paper is to use a semi-trailer axle with steering wheels, with steering angle control depending on the traffic conditions.

The aim of optimizing semi-trailer steering is to obtain the same external turning radius for the semi-trailer as for the tractor unit. For a specified steering angle of the tractor, the movement is along a circular track with a constant radius and a fixed turning center. The outermost point of the semi-trailer moves along a fixed curve. During the movement of the combination vehicle, in the case of a standard semi-trailer with rigid axles (without turning wheels), the outermost point of the semi-trailer does not move on the same track as the outermost point of the tractor; usually the radius of the track of this point is smaller. As a result, the dynamic swept path width followed by the combination vehicle may be much higher than the dynamic swept path width of the tractor or lorry alone without a trailer. In addition, there is the risk that with small turning radiuses (a sharp turn in a maneuvering area), the front outside corner of the semi-trailer can extend significantly beyond the outline of the combination vehicle and create a safety hazard.

The economic aspect is also important. In the case of a typical combination (without semi-trailer steering axles), a certain distance must be maintained between the front wall of the semi-trailer and the cab of the tractor. If this distance is too small, there could be a collision between the corner of the semi-trailer and the components of the tractor cab. The use of turning wheels on a semi-trailer requires a smaller angle of inflection of the combination vehicle (the angle between the longitudinal axis of the tractor and the longitudinal axis of the semi-trailer) during curved movement than for a typical semi-trailer. This would reduce the distance between the front wall of the semi-trailer and the tractor cab by increasing the front overhang of the semi-trailer. This would result in a greater length of usable space for the semi-trailer (volume) and reduce the aerodynamic drag of the combination vehicle [27].

## 4. Conclusions

The introduction of semi-trailer steering wheels reduces the swept path width of the vehicle. The introduction of semi-trailer steering wheels also reduces the maximum angle of kink between the longitudinal axis of the semi-trailer and the longitudinal axis of the tractor unit, so that the distance between the semi-trailer and the driver’s cab can be reduced. This solution can be used to extend the load space of the semi-trailer. Introducing a semi-trailer steering axle control algorithm would also increase the permissible length of tractor–trailer combinations [28]. Reducing the distance between the semi-trailer and the cab of the tractor unit to a minimum also leads to a reduction in aerodynamic drag, increasing operating economy and reducing emissions into the atmosphere.

The introduction of a steerable steered axle in a truck semi-trailer is much more complicated and expensive compared to the classic solution. However, such a solution can significantly improve the traction properties of the tractor–semi-trailer and improve the operational safety of the tractor–semi-trailer, where a classic vehicle set is unable to enter due to its geometric dimensions.

## Data Availability

Data are contained within the article.
